# The Disruption of NMDAR/TRPM4 Death Signaling with TwinF Interface Inhibitors: A New Pharmacological Principle for Neuroprotection

**DOI:** 10.3390/ph16081085

**Published:** 2023-07-31

**Authors:** Jing Yan, Hilmar Bading

**Affiliations:** Department of Neurobiology, Interdisciplinary Center for Neurosciences (IZN), Heidelberg University, 69120 Heidelberg, Germany

**Keywords:** neurodegeneration, NMDAR/TRPM4 death signaling, TwinF interface inhibitor, therapeutic intervention

## Abstract

With the discovery that the acquisition of toxic features by extrasynaptic NMDA receptors (NMDARs) involves their physical interaction with the non-selective cation channel, TRPM4, it has become possible to develop a new pharmacological principle for neuroprotection, namely the disruption of the NMDAR/TRPM4 death signaling complex. This can be accomplished through the expression of the TwinF domain, a 57-amino-acid-long stretch of TRPM4 that mediates its interaction with NMDARs, but also using small molecule TwinF interface (TI) inhibitors, also known as NMDAR/TRPM4 interaction interface inhibitors. Both TwinF and small molecule TI inhibitors detoxify extrasynaptic NMDARs without interfering with synaptic NMDARs, which serve important physiological functions in the brain. As the toxic signaling of extrasynaptic NMDARs contributes to a wide range of neurodegenerative conditions, TI inhibitors may offer therapeutic options for currently untreatable human neurodegenerative diseases including Amyotrophic Lateral Sclerosis, Alzheimer’s disease, and Huntington’s disease.

## 1. Introduction

Glutamate neurotoxicity, also known as excitotoxicity, refers to neuronal damage caused by the action of the neurotransmitter glutamate outside the synaptic cleft. It was first described in the retina [1] and subsequently in the brain when James Olney observed that the subcutaneous injection of monosodium glutamate induced acute neuronal death throughout the brain [2]. Glutamate neurotoxicity is associated with the loss of the structural integrity of neurons, mitochondrial dysfunction, increased production of reactive oxygen species (ROS), and deregulation of gene expression, which eventually lead to the death of neurons. Numerous studies have shown that glutamate neurotoxicity is a central part of the pathomechanism of many neurodegenerative diseases including stroke, Alzheimer’s disease (AD), Huntington’s disease (HD), and Amyotrophic Lateral Sclerosis (ALS). Therefore, huge efforts have been made in both academia and the pharmaceutical industry to investigate the molecular basis of glutamate neurotoxicity with the goal of developing effective therapeutic strategies. In the 1980s, the N-methyl-D-aspartate receptor (NMDAR) was identified as the key mediator of glutamate-induced neuronal death [3]. Later, in 2002, NMDARs located outside of synaptic contacts, so-called extrasynaptic NMDARs (eNMDARs), were found to be responsible for the toxic actions of glutamate [4], which comprise the typical pathological triad of structural disintegration, mitochondrial dysfunction, and transcriptional deregulation [5]. In the past two decades, the molecular basis of toxic eNMDAR signaling has been investigated in depth, and in 2020, it led to the discovery of the death signaling complex and a new pharmacological principle for providing neuroprotection [6]. In this review, we focus on the molecular basis of eNMDAR-mediated excitotoxicity and summarize pre-clinical and clinical studies that have employed NMDAR antagonists in an effort to treat neurodegenerative diseases. Finally, we describe a conceptually new approach for therapeutic interventions.

## 2. Deregulation of Glutamate Homeostasis in Neurodegeneration

**Figure 1:**
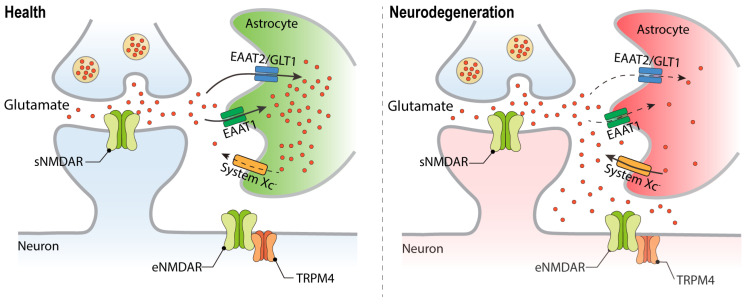
Deregulation of glutamate homeostasis in neurodegeneration. Faulty glutamate re-uptake systems in neurodegenerative diseases lead to elevated glutamate levels outside the synaptic contacts and the stimulation of the extrasynaptically localized NMDAR/TRPM4 complex. Excitatory amino acid transporter (EAAT); System XC, amino acid antiporter that mediates the exchange of extracellular L-cystine and intracellular glutamate.

Glutamate is one of the most abundant neurotransmitters in the central nervous system and is the principal mediator of excitatory synaptic transmission in the mammalian brain. Upon depolarization of neurons, glutamate is released from nerve terminals into the synaptic cleft and activates glutamate receptors in the synapse. Efficient excitatory amino acid transporters (EAATs) mediate the re-uptake of glutamate into neurons and glial cells, thereby preventing glutamate leakage into the extrasynaptic space [7,8,9,10]. Many acute and chronic neurodegenerative disorders are characterized by deficits in glutamate uptake systems, which causes extracellular glutamate concentrations to increase to neurotoxic levels. Hypoxic ischemic conditions can further exacerbate deregulation of glutamate homeostasis through enhanced extrasynaptic glutamate release via the cystine/glutamate antiporter, System X_C_ [11] (Figure 1). Thus, while in healthy condition, the action of glutamate is restricted to the synapse, in neurodegenerative diseases, glutamate receptors located outside synaptic contacts are being activated. Particularly relevant in the context of glutamate neurotoxicity are eNMDARs (Figure 1; and see below).

## 3. Glutamate Neurotoxicity and NMDA Receptors

Glutamate acts on two types of glutamate receptors: ionotropic glutamate receptors (iGluRs) and metabotropic glutamate receptors (mGluRs). The iGluRs, which include α-amino-3-hydroxy-5-methyl-4-isoxazole propionic acid receptors (AMPARs), kainate receptors, and NMDARs, are glutamate-gated cation channels that—upon their activation—allow for ion flux across the plasma membrane. In contrast, mGluRs belong to the family of G-protein-coupled receptors that are linked to either the cAMP or phosphatidylinositol signaling pathways. Although all types of glutamate receptors have been linked to glutamate neurotoxicity, the NMDAR is generally considered the major player in excitotoxicity. NMDARs consist of four subunits in various subtype configurations. Typically, NMDARs include two mandatory GluN1 subunits and either two GluN2 or GluN3 subunits. Four subtypes of GluN2 (GluN2A, GluN2B, GluN2C, and GluN2D) and two subtypes of GluN3 (GluN3A and GluN3B) subunits are known. Despite differences in their molecular weight and biochemical properties, all three types of NMDAR subunits share common structural features, including an extracellular domain that binds agonists, a transmembrane domain, and a C-terminal domain that regulates intracellular trafficking, membrane insertion, protein–protein interactions, and downstream signaling [12,13].

Although NMDARs are the key mediators of excitotoxic neuronal death, they are also critical for neuronal survival, synaptic transmission, and memory formation [14,15,16]. Accordingly, despite their neuroprotective potential in animal models of neurological disorders, including stroke, glaucoma, AD, HD, and ALS, the application of NMDAR antagonists in patients is limited. Indeed, as classical NMDAR antagonists such as MK801 failed to make it to clinical trials, research aimed at addressing the toxic functions of NMDARs shifted towards the generation of subtype-specific antagonists and in particular towards blockers of GluN2B-containing NMDARs, which are considered to be the predominant NMDAR subunits driving glutamate neurotoxicity [17,18].

Ifenprodil (NP-120) and its analog eliprodil (SL-82.0715) were developed as selective antagonists for GluN2B-containing NMDARs, and they have shown neuroprotective efficacy in animal models of stroke [19,20,21]. There have been several clinical studies examining the efficacy of eliprodil for treating stroke. No results have been published; however, according to its manufacturer Sanofi-Synthélabo, a phase III clinical trial failed. Although no side effects were observed with the treatment regimen [22], both ifenprodil and eliprodil are also known to act as antagonists of α1-adrenergic receptors, serotonin receptors, and calcium channels [23,24,25,26]. Accordingly, their effectiveness may be compromised due to potential interference with the cardiovascular system. To address this issue, several “second generation” ifenprodil analogs, including traxoprodil (CP-101606; Pfizer), were developed. CP-101606 was demonstrated to have greater selectivity for GluN2B receptor subtypes over other targets and to provide robust neuroprotection in various animal models of stroke [27,28,29]. In a double-blind, placebo-controlled study of CP-101606 in patients with mild or moderate traumatic brain injury, no psychotropic effects were found, and it was well tolerated [30]. However, although the Neurobehavioral Rating Scale score of all subjects improved compared to a pre-dosing baseline, no significant differences were observed either with respect to the type of head injury, or to the treatment—drug or placebo—received. In another open-label study with 30 patients with severe traumatic brain injury, a 72 h infusion of CP-101606 was well tolerated; it effectively penetrated the cerebrospinal fluid and brain and improved outcomes in brain-injured patients, with longer infusions showing better average outcomes [31]. However, the development of CP-101606 was discontinued due to potential cardiovascular toxicity, especially of prolonged QT interval [32].

Several lines of evidence have challenged the hypothesis that GluN2B-containing NMDARs are the predominant drivers of glutamate neurotoxicity, and instead implicate both GluN2A and GluN2B as mediators of glutamate-induced neuronal damage. In non-neuronal cell lines such as human embryonic kidney (HEK) 293 cells, for example, expression of functional NMDARs leads to severe cell damage and death regardless of whether NMDARs contain GluN2A or GluN2B subunits [33,34]. Further, GluN2B receptors are expressed earlier in the development of neurons—both in vitro and in vivo—compared to GluN2A receptors. However, ifenprodil shows significant neuroprotection only during early developmental stages, but fails to protect neurons from glutamate neurotoxicity at a more mature stages, indicating that, in more developed neurons expressing both receptor subtypes, both GluN2A and GluN2B mediate glutamate toxicity [35,36].

As an alternative to the subunit hypothesis dictating that GluN2B-containing NMDARs mediate glutamate neurotoxicity, it was proposed in 2002 by Bading and colleagues that NMDARs localized outside the synaptic cleft are the predominant triggers of excitotoxicity [4]. It is now generally accepted that the subcellular location of NMDARs is critical for the outcome of their stimulation. The activation of synaptic NMDARs (sNMDARs) during action potential bursting has been shown to trigger downstream signaling pathways involving the CaMKIV- and CREB-dependent expression of immediate early genes (IEGs), and ERK signaling, leading to enhanced neuronal survival and synaptic plasticity. In striking contrast, the activation of extrasynaptic NMDARs (eNMDARs) results in the suppression of CREB signaling, inactivation of ERK, mitochondrial dysfunction, and ultimately cell death [37,38]. Given that MK-801 is an open-channel NMDAR blocker, sNMDARs can be selectively inhibited in a paradigm that involves the activation of sNMDARs via synaptic activity in the presence of MK801, leaving eNMDARs unblocked. After the washout of MK801, the latter receptors can be subsequently stimulated via bath-applied glutamate or NMDA. This protocol enables the selective activation of eNMDARs and the separate study of the function of sNMDARs and eNMDARs [4].

## 4. Neuroprotectants Targeting Glutamate Neurotoxicity

Increased glutamate levels and the activation of eNMDARs represent a critical point of convergence in the pathomechanism of many neurodegenerative diseases. Several drugs targeting this mechanism have been developed and tested in clinical trials, but only two have been approved by the Food and Drug Administration (FDA) and the European Medicines Agency (EMA): riluzole for ALS and memantine for moderate to severe AD. While these medications have also been investigated for other neurodegenerative diseases, the results have been largely disappointing. Here, we provide an overview of the preclinical and clinical outcomes of riluzole and memantine across a broad range of neurodegenerative diseases (see also Table 1).

## 5. Riluzole

Riluzole (2-Amino-6-trifluoromethoxy benzothiazole) was developed by the French chemical company Rhône-Poulenc Rorer (now Sanofi) in the 1980s as a possible antagonist of excitatory amino acids including glutamate [39,40]. The exact mode of action of riluzole is unclear, but it is believed to provide neuroprotection by reducing the release and increasing the uptake of glutamate in the brain and spinal cord [41,42,43,44]. Riluzole can also block NMDARs in Xenopus oocytes in a reversible and competitive manner [45] as well as human muscle acetylcholine receptors [46,47], suggesting other possible neuroprotective mechanisms of action. Riluzole is also an effective blocker for both tetrodotoxin (TTX)-sensitive and TTX-resistant sodium channels; it can block the inactivated sodium channel of damaged neurons under ischemic conditions, which may provide neuroprotection [48].

Given its ability to prevent glutamate-mediated neuronal death, the therapeutic potential of riluzole was evaluated with ALS patients starting in the early 1990s, only a few years after its discovery [49,50,51]. The first trial comprised a total of 155 patients. Riluzole (100 mg/day) significantly increased the survival rate from 58% to 74% after 12 months of treatment. The deterioration of muscle strength was also slowed by riluzole treatment [49]. A second trial with 959 ALS patients was conducted a short time later to evaluate the efficacy of riluzole at different doses (50, 100, or 200 mg/day). Although no functional improvements were observed in this trial—including measures of muscle strength—all three riluzole doses reduced the risk of death or tracheostomy after 12 and 18 months, with the greatest reduction observed in patients receiving higher doses [51]. Not surprisingly, as riluzole has many biological targets, several adverse effects were documented, including dizziness, gastrointestinal disorders, increased alanine aminotransferase, and low hemoglobin levels. Nonetheless, as the results of these studies demonstrated a modest benefit in extending the lifespan of ALS patients, riluzole was approved for the treatment of ALS by the FDA in 1995 and by the EMA one year later.

Given its potential to reduce glutamate neurotoxicity, riluzole has been evaluated for its potential to treat other neurodegenerative diseases, including HD, PD, and AD (Table 1). In a preclinical study, riluzole extended the survival time of R6/2 HD model mice, and to reduce the severity of intranuclear inclusions in their striata [52]. In a phase III clinical trial involving 537 HD patients given a 100 mg daily dose for three years, however, no neuroprotective or other beneficial effects were observed [53]. Similarly, although riluzole had provided neuroprotection in cellular and animal models of PD [54,55], it failed to improve survival or deterioration rates in PD patients [56]. In contrast to these somewhat disappointing results, riluzole has demonstrated the potential to treat AD in preclinical settings. When administered to mouse models of AD, riluzole was able to rescue the ageing- and AD-related gene expression profiles, cognitive deficits, and memory deficits [57,58,59,60,61,62]. A recent phase II trial with AD patients revealed that cerebral glucose metabolism, a well-established biomarker for AD, was significantly better preserved in riluzole-treated patients than in the placebo group [63]. Despite its promise as revealed by this study, a higher-powered trial of longer duration is necessary in order to validate the potential therapeutic effects of riluzole in AD. Preclinical studies have also revealed that riluzole shows potential in the treatment of ischemic stroke and glaucoma: it showed efficacy in preventing brain damage and in delaying retinal ganglion cell degeneration in mouse models of ischemic stroke and glaucoma, respectively [64,65,66,67]. To our knowledge, however, there have not yet been any clinical trials investigating the therapeutic potential of riluzole in these diseases.

## 6. Memantine

In the early 1960s, Eli Lilly synthesized memantine hydrochloride with the aim of developing an antidiabetic drug [68]. Although ineffective for reducing elevated blood sugar levels, memantine was later found, in 1989, to be a clinically well-tolerated NMDAR antagonist [69]. Notably, unlike several other NMDAR antagonists, memantine exhibits a strong voltage dependency and rapid unblocking kinetics, characteristics which allow it—when used at low doses—to preferentially affect tonically activated eNMDARs while leaving the normal physiological functions mediated by sNMDARs largely unaffected [70]. Memantine is not, however, a pure NMDAR antagonist: it also acts as a non-competitive antagonist for type 3 serotonin receptors and alpha7 nicotinic acetylcholine receptors, and is an agonist of dopamine D2 receptors and of sigma 1 receptors [71,72,73,74,75].

Merz Pharma (Germany) initiated investigations into the therapeutic potential of memantine for treating dementia in 1989 [76]. Subsequently, a randomized, double-blind, placebo-controlled clinical trial published in 2003 found that memantine significantly improved cognitive function and daily living activities in patients with moderate to severe AD [77]. The study involved 252 patients who were treated with either memantine or placebo for 28 weeks. Based on this study, memantine was approved by the FDA for the treatment of moderate to severe AD. Later, memantine was used in conjunction with donepezil, an acetylcholinesterase (AChE) inhibitor commonly prescribed for treating AD. Compared to donepezil alone, this combination has been shown to lead to significantly improved outcomes on measures of cognition, daily living activities, and global outcomes, and to reductions in agitation/aggression, irritability, and appetite/eating disturbances in patients with moderate to severe AD [78,79]. However, another trial failed to demonstrate a significant difference in the effectiveness of donepezil and memantine together compared to either treatment alone [80]. Nonetheless, as memantine has a different mechanism of action, its administration in combination with AChe inhibitors may offer significant benefits to some AD patients.

The potential effectiveness of memantine in the treatment of neurological disorders has been explored for several disorders other than AD, including HD, glaucoma, ALS, and multiple sclerosis. In the striatum of the YAC128 mouse model of HD, there was increased expression of eNMDARs and increased eNMDAR currents, as well as reduced CREB activation and increased cell death [81]. The chronic treatment of these mice with memantine was found to restore nuclear CREB phosphorylation and to improve motor learning, suggesting that memantine may provide therapeutic benefits in HD [81]. To investigate the effectiveness of memantine in human patients, a two-year open-label multicenter trial was conducted with 27 HD patients who received up to 30 mg/day of memantine, and demonstrated that memantine treatment slowed disease progression [82]. Another small pilot study revealed that a daily dose of 20 mg of memantine led to significant improvements in motor symptoms—particularly chorea—but did not improve cognitive, behavioral, functional, or independence ratings in treated patients [83]. However, as placebo controls were missing in both trials, and no follow-up studies have been conducted, it is difficult to fully assess the therapeutic benefits of memantine in HD.

As concerns glaucoma, the effectiveness of memantine has been well documented in both monkey and rat models of glaucoma [84,85]. While memantine did not affect the normal function of the retina as assessed with electroretinogram (ERG) and visually evoked cortical potential (VECP) recordings, its administration did significantly prevent the reduction of VECP responses in a monkey model of glaucoma and was associated with diminished RGC loss in a rat glaucoma model [85]. Moreover, topically applied memantine significantly reduced RGC loss in a rodent model of ocular hypertension [86]. Despite these promising pre-clinical results, the clinical transition of memantine as a glaucoma treatment failed to prevent glaucomatous progression in two phase III studies involving more than 2000 glaucoma patients [87]. Notably, although it is well documented that memantine can protect RGCs in glaucoma, it is unknown whether memantine also protects RGC axons. As axons of RGCs are also affected in glaucoma patients, a failure to protect them may explain why memantine has failed in clinical trials to improve outcomes for glaucoma patients [88].

Memantine was reported in 2005 to extend the survival of the SOD1^G93A^ mouse model of ALS [89]. However, a randomized controlled trial conducted in 2010 and involving 63 patients failed to show any beneficial effect of memantine in ALS [90]. In MS patients, memantine even worsened the neurological symptoms [91]. Thus, although memantine preferentially blocks eNMDARs and is FDA approved for moderate to severe AD, it has not yet proved beneficial in other neurodegenerative disorders (Table 1).

## 7. NMDAR Interacting Proteins

Protein–protein interactions play a vital role in NMDAR-mediated downstream signaling [96,97]. NMDAR interacting proteins not only participate in physiological processes but also contribute to excitotoxicity. An important observation that subsequently helped guide the development of effective neuroprotective strategies (see below) was the discovery, in 2002, that the location of NMDARs and their interacting proteins determine the outcome of NMDAR stimulation: activation of synaptically localized NMDARs promotes neuronal survival and regulates synaptic plasticity, whereas the activation of NMDARs located extrasynaptically promotes death signaling and kills neurons [4].

The first reported NMDAR-linked protein complex with a possible role in mediating glutamate toxicity comprises neuronal nitric-oxide synthase (NOS), the NMDAR subunit GluN2B, and post-synaptic density 95 protein (PSD95) [98]. The disruption of GluN2B-PSD95 interactions by means of peptides or proteins that harbor nine specific amino acids derived from the C-terminus of GluN2B protected against excitotoxicity-induced neuronal death in vitro and against brain damage in mouse model of ischemia [99]. Building on these findings, PSD-95-targeting strategies were developed, including a PSD-95 inhibitor to treat stroke in the hydrocephalic primate brain [100]. A second protein reported to interact with the C-terminal domain of the GluN2B subunit is the death-associated protein kinase 1 (DAPK1) [101]. Similar to PSD95, the interruption of the GluN2B-DAPK1 interaction via an interfering peptide or via the genetic deletion of DAPK1 provided protection against brain damage in ischemic stroke [101]. Despite this original finding, however, the role of DAPK1 in excitotoxicity remains controversial [102].

**Figure 2:**
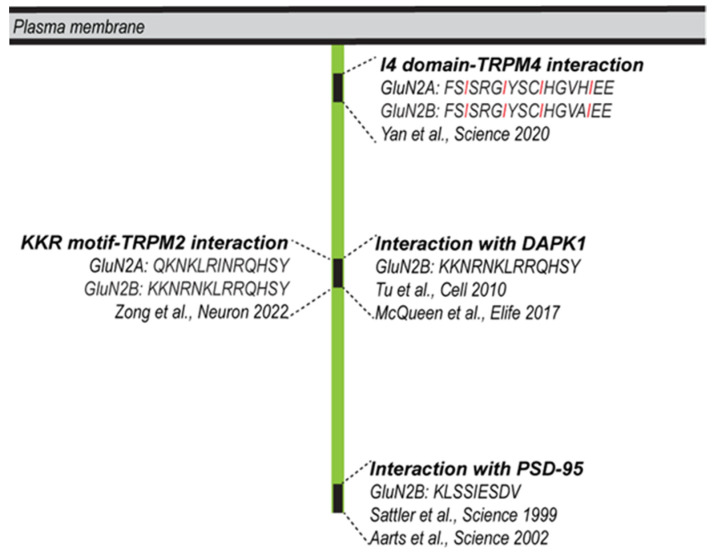
Protein–protein interaction domains within the cytoplasmic portions of GluN2A/2B with possible roles in cell death. GluN2A/2B can interact with PSD95, Sattler et al., 1999 [98], Aarts et al., 2002 [99]; DAPK1, Tu et al., 2010 [101], McQueen et al., 2017 [102]; TRPM4, Yan et al., 2020 [6]; TRPM2, Zong et al., 2022 [103]. The domain of GluN2B that interacts with TRPM2 can also interact with DAPK1. The four regularly spaced isoleucines of the I4 domain are highlighted in red.

In recent years, two NMDAR-interacting proteins of the transient receptor potential melastatin subfamilies, TRPM2 and TRPM4, have been linked to cell death [6,103]. TRPM2 is a calcium-permeable channel that is activated through intracellular calcium and ADP ribose and also regulated via oxidative stress. TRPM2 contributes to brain injury in ischemic stroke, possibly due to its ability to regulate NMDAR trafficking [103]. In particular, TRPM2-NMDAR coupling is enhanced subsequent to ischemic stroke, resulting in a Protein Kinase C gamma (PKC-γ)-dependent increase in NMDAR expression at the cell surface. The ‘EE_3_’ motif of TRPM2 and the ‘KKR’ motif of the NMDAR mediate their interaction, which—when disrupted using an EE_3_ motif peptide—can protect cultured neurons from oxygen glucose deprivation (OGD)-induced neuronal death in vitro and can reduce brain damage following ischemic stroke. Interestingly, the domain of GluN2B that harbors the TRPM2 binding ‘KKR’ motif also interacts with DAPK1 [101] (Figure 2). If and how the TRPM2-NMDAR or the DAPK1-NMDAR interactions contribute to neurodegenerative diseases other than stroke remains to be investigated.

## 8. The NMDAR/TRPM4 Death Signaling Complex

While the interaction between TRPM2 and NMDAR seems to be induced under excitotoxic conditions, TRPM4 and NMDAR form a stable complex under physiological conditions [6,104]. TRPM4 is a non-selective monovalent cation channel, which is activated by intracellular calcium and inhibited by intracellular ATP [105,106]. The genetic deletion or pharmacological inhibition of TRPM4 can provide neuroprotection in an experimental autoimmune encephalomyelitis (EAE) mouse model [107]. Moreover, primary neuronal cultures derived from TRPM4 knock out mice as well as wild-type mouse neurons in which TRPM4 was knocked down using RNA interference technology are protected from glutamate neurotoxicity [107]. Moreover, the pharmacological inhibition of TRPM4 protects rodents from both ischemic and hemorrhagic stroke [108,109,110,111,112,113,114]. In light of our understanding that eNMDARs are the principal mediators of glutamate neurotoxicity whereas sNMDARs promote survival and plasticity [4], it is perhaps unsurprising that TRPM4-NMDAR interactions seem to take place at extrasynaptic locations [6]. Accordingly, the disruption of the eNMDAR/TRPM4 death signaling complex detoxifies eNMDARs, providing a mechanistic framework for the generation of a new type of neuroprotectant.

## 9. A New Pharmacological Principle in Neuroprotection: Disruption of the NMDAR/TRPM4 Death Signaling Complex

NMDARs located extrasynaptically gain toxicity through their interaction with TRPM4 [6]. The mapping of the domains that mediate the interaction of TRPM4 with the NMDAR has guided the development of both the recombinant and small molecule inhibitors of their interaction interface. The recombinant interface inhibitor is the TRPM4 interface itself, i.e., the domain of TRPM4 that makes contact with the NMDAR. This domain is a 57-amino-acid-long cytosolic portion of TRPM4, named TwinF, that interacts with an 18-amino-acid-long domain of the NMDAR subunits GluN2A and GluN2B, named I4 [6]. Small molecule interface inhibitors were identified in a computer assisted, structure-based screening for compounds that interact with the core region of TwinF. The first two prototype small molecules TwinF interface (TI) inhibitors were compound 8 and compound 19. TwinF and TI inhibitors (previously termed ‘NMDAR/TRPM4 interaction interface inhibitors’) offer robust protection against NMDA- and OGD-induced excitotoxicity in primary neurons, and reduce both NMDA-induced RGC loss in mice and brain damage following middle cerebral artery occlusion [6]. However, as they do not affect the function of sNMDARs, but instead seem to specifically target the toxic signaling component of eNMDAR activity. These inhibitors represent a new and potentially powerful therapeutic concept for treating neurodegenerative diseases involving glutamate neurotoxicity.

## 10. TI Inhibitors

**Figure 3:**
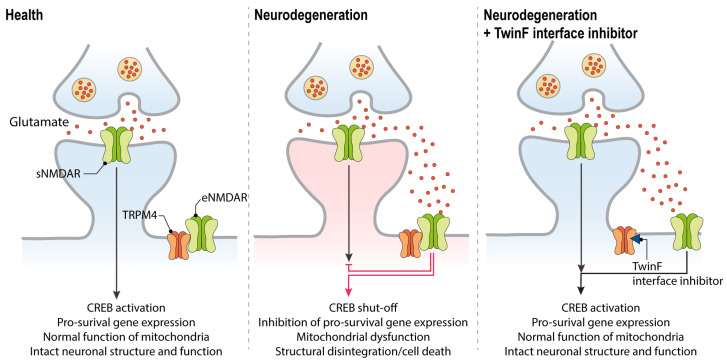
The therapeutic action of TwinF interface (TI) inhibitors in neurodegenerative diseases. In the healthy brain, synaptic NMDAR activation promotes CREB activation and pro-survival gene expression (**left panel**). In neurodegenerative diseases, glutamate levels outside the synapse increase and result in the activation of the eNMDAR/TRPM4 complex. This triggers the typical pathological triad of glutamate neurotoxicity, which consists of CREB shut-off and the deregulation of gene expression, mitochondrial dysfunction, and loss of structural integrity, eventually leading to neuronal cell death (**middle panel**). By disrupting the NMDAR/TRPM4 complex, TI inhibitors can abolish toxic signaling, rescue CREB-mediated transcription, and restore the normal functioning of mitochondria (**right panel**). Glial cells that are primarily responsible for glutamate re-uptake are not depicted (see Figure 1).

Thus far, TI inhibitors exhibit superiority over other neuroprotective compounds because, in addition to protecting against cell death and mitochondrial dysfunction, they can also revert the CREB shut-off associated with excitotoxicity. Accordingly, TI inhibitors can rescue the deregulated gene expression associated with glutamate neurotoxicity [4,5], and convert an eNMDAR-activating stimulus into a transcription-promoting one [6,115]. More specifically, an excitotoxic stimulus applied to cultured primary neurons will result in the inhibition of synaptic-activity-induced CREB phosphorylation and CREB-mediated gene expression. Among the genes whose expression is thereby inhibited are *Bdnf*, *Npas4*, and *cFos*, all genes that are otherwise induced upon synaptic activity and sNMDAR activation, and which serve important functions in the nervous system [15]. Classical blockers of NMDARs do protect against the toxic consequences of excitotoxic stimuli, but they also inhibit the synaptic-activity-driven, CREB-mediated gene expression that is vital for a healthy brain. In contrast, by separating eNMDARs from TRPM4, TI inhibitors not only detoxify eNMDARs, but also enable them to function in a similar manner as sNMDAR. This mechanism of action—by which both the physiological functions of sNMDARs are preserved and by which excitotoxic stimuli acting on eNMDARs are converted into beneficial signals [6]—holds great promise for therapeutic interventions (Figure 3).

## 11. Wide Range of Possible Therapeutic Applications of TI Inhibitors

**Figure 4:**
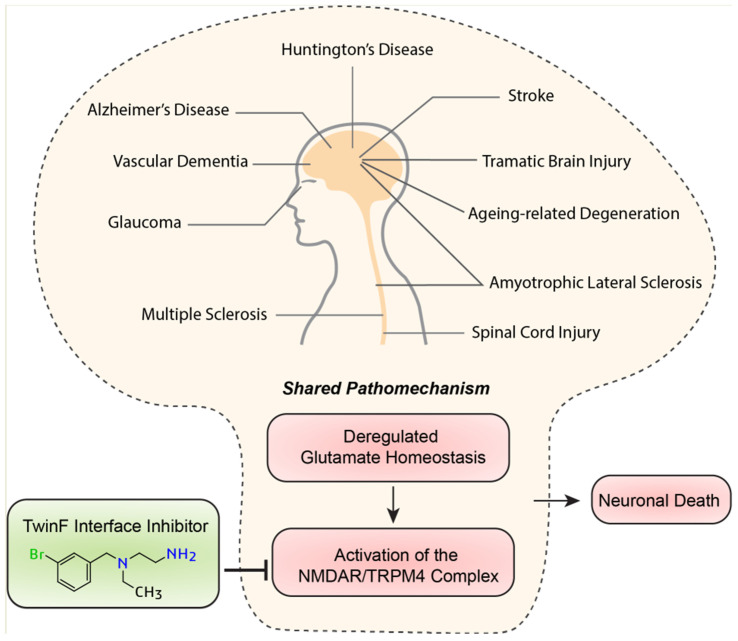
Different diseases—shared pathomechanism. The deregulation of glutamate homeostasis activating toxic signaling via the NMDAR/TRPM4 complex is a converging point in the cell pathology of many neurodegenerative diseases. TI inhibitors may be widely applicable as neuroprotective therapeutics.

Signaling induced via activated eNMDARs has emerged as a central component of the pathomechanism for a wide range of acute and chronic neurodegenerative conditions, including ALS, AD, HD, glaucoma, vascular dementia, stroke, traumatic brain or spinal cord injury, and ageing-related degeneration [5,37] (Figure 4). Even the development of chronic neuropathic pain may involve toxic eNMDAR signaling and the subsequent degeneration of neurons [116]. Indeed, the cell pathology common to virtually all neurodegenerative conditions is highly reminiscent of the typical pathological triad triggered by the activation of eNMDARs: loss of structural integrity (i.e., the loss of synapses and dendrites), mitochondrial dysfunction (i.e., the breakdown of the mitochondrial membrane potential, metabolic/energy insufficiency, and increased production of reactive oxygen species), and transcriptional deregulation (i.e., CREB shut-off and reduced expression of activity-regulated neurotrophic/neuroprotective genes) [4,5,38]. One reason for the convergence of different pathomechanisms on toxic eNMDAR signaling is that virtually all neurodegenerative conditions are burdened with faulty or deregulated glutamate uptake systems, resulting in the leakage of synaptically released glutamate and a subsequent rise in glutamate levels at extrasynaptic locations [7,11,117,118,119]. Deregulated glutamate homeostasis is further enhanced via neuroinflammatory responses and a leaky blood–brain barrier, both of which are often associated with degenerative processes in the brain. TI inhibitors would not fix aberrant neurotoxic glutamate levels, but they do have the potential to break the disease process by detoxifying eNMDAR signaling, and thereby restoring normal mitochondrial function, maintaining proper gene regulation, and preserving neurons’ structural integrity. In sum, TI inhibitors hold great potential as broad-spectrum neurotherapeutics, raising our hopes that currently untreatable human neurodegenerative diseases may become treatable.

## Figures and Tables

**Table 1:** Summary of clinical trials with riluzole and memantine. UHDRS: unified Huntington’s disease rating scale; ALSFRS: ALS functional rating scale; PPS: Parkinson Plus Syndromes; NAA: N-acetylaspartate; CIBIC-Plus: Clinician’s Interview-Based Impression of Change Plus Caregiver Input; ADCS-ADLsev: Alzheimer’s Disease Cooperative Study Activities of Daily Living Inventory modified for severe dementia. O.d.: once a day. B.i.d.: twice a day. * In this study, the dose of memantine was not specified by increasing to a maximum of 30 mg/day, according to the tolerance of the individual patient. N.D.: not determined.

	Disease	Patients Complete/Total	Details	Evaluation (Primary Endpoint)	Dose	Phase	Outcome	Ref.
RILUZOLE	ALS	155/155	Randomized, placebo-controlled, double-blind, 1-year trial	Survival and Norris scales	100 mg o.d.	N.D.	Positive	[49]
	ALS	959/959	Randomized, placebo-controlled, double-blind, 12- or 18-month trial	Survival	50, 100, or 200 mg. o.d.	N.D.	Positive	[51]
	AD	42/50	Randomized, placebo-controlled, double-blind, 6-month trial	Cerebral glucose metabolism and NAA	50 mg b.i.d.	Phase 2 NCT01703117	Positive	[63]
	HD	8/8	Open-label, 6-week trial	UHDRS	50 mg b.i.d.	N.D.	Negative	[92]
	HD	7/9	Open-label, 12-month trial	UHDRS	50 mg b.i.d.	N.D.	Benefits at 3 months, but no longer at 12 months	[93]
	HD	56/63	Randomized, placebo-controlled, double-blind, 8-week trial	UHDRS	100 or 200 mg o.d.	N.D.	Negative	[94]
	HD	379/537	Randomized, placebo-controlled, double-blind, 3-year trial	UHDRS	50 mg b.i.d.	Phase 3 NCT00277602	Negative	[53]
	PPS	760/767	Randomized, placebo-controlled, double-blind, 3-year trial	Survival	50–200 mg o.d.	Phase 3 NCT00211224	Negative	[56]
	MS	198/223	Randomized, placebo-controlled, double-blind, 96-week trial	Change in brain volume	50 mg o.d. until week 4, then b.i.d.	Phase 2b NCT01910259	Negative	[95]
MEMANTINE	AD (moderate to severe)	181/252	Randomized, placebo-controlled, double-blind, 28-week trial	CIBIC-Plus ADCS-ADLsev	20 mg o.d.	N.D.	Positive	[77]
	Glaucoma	1877/2298	Randomized, placebo-controlled, double-blind, 2-year trials	Visual field progression	10 and 20 mg o.d.	Phase 3 NCT00141882 NCT00168350	Negative	[87]
	HD	9/12	Open-label, 3-month pilot study	UHDRS	5–20 mg o.d.	N.D.	Positive/negative	[83]
	HD	27/27	Open-label, 2-year pilot study	Clinical assessment	30 mg *, o.d.	N.D.	Positive	[82]
	ALS	50/63	Randomized, placebo-controlled, double-blind, 12-month trial	change in ALSFRS	20 mg o.d.	Phase 2/3 NCT00353665	Negative	[90]
	MS	19/50	Randomized, placebo-controlled, double-blind, 12-month trial	Verbal memory	30 mg o.d.	NCT00638833	Neurologic symptoms worsened	[91]

## Data Availability

No new data were created or analyzed in this study. Data sharing is not applicable to this article.
